# Kidney failure in Bardet–Biedl syndrome

**DOI:** 10.1111/cge.14119

**Published:** 2022-03-13

**Authors:** Jennifer R. Meyer, Anthony D. Krentz, Richard L. Berg, Jesse G. Richardson, Jeremy Pomeroy, Scott J. Hebbring, Robert M. Haws

**Affiliations:** ^1^ School of Medicine and Public Health University of Wisconsin Madison Wisconsin USA; ^2^ PreventionGenetics Marshfield Wisconsin USA; ^3^ Office of Research Computing and Analytics Marshfield Clinic Research Institute Marshfield Wisconsin USA; ^4^ Clinical Research Center Marshfield Clinic Research Institute Marshfield Wisconsin USA; ^5^ Precision Medicine Research Marshfield Clinic Research Institute Marshfield Wisconsin USA; ^6^ Department of Pediatrics Marshfield Clinic Health System Marshfield Wisconsin USA; ^7^ Present address: M Health Fairview Maternal‐Fetal Health Minneapolis Minnesota USA

**Keywords:** Bardet–Biedl syndrome, chronic kidney disease, ciliopathies, genetic association studies, urogenital abnormalities

## Abstract

The aim of this study was to explore kidney failure (KF) in Bardet–Biedl syndrome (BBS), focusing on high‐risk gene variants, demographics, and morbidity. We employed the Clinical Registry Investigating BBS (CRIBBS) to identify 44 (7.2%) individuals with KF out of 607 subjects. Molecularly confirmed BBS was identified in 37 KF subjects and 364 CRIBBS registrants. KF was concomitant with recessive causal variants in 12 genes, with *BBS10* the most predominant causal gene (26.6%), while disease penetrance was highest in *SDCCAG8* (100%). Two truncating variants were present in 67.6% of KF cases. KF incidence was increased in genes not belonging to the BBSome or chaperonin‐like genes (*p* < 0.001), including *TTC21B*, a new candidate BBS gene. Median age of KF was 12.5 years, with the vast majority of KF occurring by 30 years (86.3%). Females were disproportionately affected (77.3%). Diverse uropathies were identified, but were not more common in the KF group (*p* = 0.672). Kidney failure was evident in 11 of 15 (73.3%) deaths outside infancy. We conclude that KF poses a significant risk for premature morbidity in BBS. Risk factors for KF include female sex, truncating variants, and genes other than BBSome/chaperonin‐like genes highlighting the value of comprehensive genetic investigation.

## Graphical abstract

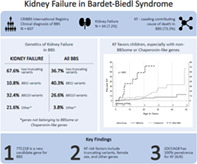

## 1 INTRODUCTION

Kidney failure (KF) is a primary cause of premature death in Bardet–Biedl syndrome (BBS, OMIM 209900), a rare, autosomal recessive, and renal ciliopathy.^1^ Despite significant advances in identifying 26 disease‐causing genes,^2^, ^3^, ^4^ recognition of disruption of the assembly and function of the primary cilium as a central mechanism underlying BBS,^5^, ^6^ and current efforts advancing translational therapies for two primary features of BBS (obesity^7^ and retinal degeneration^8^), there remains a significant gap in understanding the genetic underpinnings of KF.

Manifestations of kidney disease are highly variable in BBS. Prenatal imaging may identify nephromegaly, with increased renal echogenicity and cystic changes often confused with autosomal recessive polycystic kidney disease.^9^ Perinatal death associated with hydrops fetalis and absent kidney function has been observed.^9^, ^10^Fewer than 10% of children with BBS have been noted to have severely impaired kidney function, while milder stages of chronic kidney disease (CKD) are reported in one‐third of children, and normal or near normal renal function in over 50%.^11^ The prevalence of severe CKD in adults with BBS is reported in 8%–16%,^1^, ^11^, ^12^, ^13^ while the presence of mild or moderate CKD (CKD2‐3) is similar to that in children. Hyposthenuria, reduced urine solute concentrating capacity, resulting in polyuria and polydipsia is observed in all stages of CKD, and its presence may be a warning sign for poor renal outcome.^12^, ^14^, ^15^ Renal biopsy is infrequently performed, but renal dysplasia and chronic tubulointerstitial nephropathy are typically identified in individuals with CKD.^10^, ^12^ Structural genitourinary anomalies are common in BBS and include persistent fetal lobulation, parenchymal and calyceal cysts, hydronephrosis, renal agenesis, horseshoe kidney, vesicoureteral reflux, vaginal atresia, and urogenital sinus.^11^, ^12^, ^16^ Urinary tract infection is common, while hematuria and proteinuria are absent or generally mild.^1^, ^10^, ^12^ For those individuals in whom KF occurs, its onset is typically insidious and transpires in the first or second decade of life. Renal transplantation has been shown to result in favorable outcomes for patients with BBS and KF.^17^

The correlation of the BBS renal phenotype with the diverse BBS genes may yield important insight in risk factors for KF. A previous study identified an increased prevalence of severe kidney disease defined as an estimated glomerular filtration rate (eGFR) < 45 ml/min/1.73 m^2^ in individuals with *BBS2*, *BBS10*, and *BBS12* compared to individuals with *BBS1*.^11^ In the same study, homozygous and compound heterozygous individuals with truncating variants were more likely to be associated with severe kidney disease than those with two missense variants. More severe CKD was observed in a French adult cohort (>16 years) with *MKKS*, *BBS10*, and *BBS12*.^13^ These three genes comprise the chaperonin‐like genes that encode proteins responsible for mediating assembly of an eight‐subunit protein particle, designated as the BBSome (*BBS1*, *BBS2*, *BBS4*, *BBS5*, *BBS7*, *TTC8/BBS8*, *BBS9*, and *BBIP1/BBS18*).^18^, ^19^, ^20^ The BBSome works in concert with intraflagellar transport machinery mediating trafficking of transmembrane cargos into and out of the cilia.^21^ Chaperonin‐like genes have been associated with a more severe phenotype in some reports.^2^, ^19^, ^20^ Genes designated in this report as “Other BBS” genes consist of genes identified in individuals with BBS that are expected vital to ciliogenesis and ciliary function but not members of the BBSome or chaperonin‐like proteins.^21^, ^22^, ^23^, ^24^, ^25^ The genotype:phenotype correlation of the other BBS genes and KF risk is largely unexplored in BBS.^24^, ^25^, ^26^ The severity of hyposthenuria correlates with both truncating genetic variants and reduced eGFR, suggesting opportunities to explore unknown genetic and pathogenic mechanisms for KF.^14^, ^15^Furthermore, the genetic correlations of genitourinary anomalies in BBS and KF have not been systematically examined.

While KF occurs in only a minority of individuals with BBS and favors children under 20 years‐of‐age,^11^ clinicians currently have limited ability to identify children at highest risk of progressing to KF.^10^, ^11^ Further characterization of genotype–phenotype relationships would allow clinicians to better predict the renal disease course of BBS patients and provide families with more tailored anticipatory guidance.^27^ Additionally, improved understanding of genotype–phenotype correlations in this genetically heterogeneous disease may lead to increased understanding of the molecular pathogenesis of KF in BBS.^2^, ^6^, ^15^

In this study, we investigated the incidence of KF and genetic predictors of kidney failure in BBS. A secondary aim was to explore BBS variants based on their role in primary cilia assembly and function (BBSome, chaperonin‐like, other BBS genes) and the significance of truncating variants in KF disease expression. We report *TTC21B*, a new candidate BBS gene previously identified in other ciliopathies.^25^ The study used an international natural history database to characterize sex and age characteristics of KF. We examined the incidence and diversity of lower genitourinary (GU) anomalies that may contribute to the risk of KF. Furthermore, we report the importance of KF as a contributing risk for premature mortality in individuals with BBS.

## 2 MATERIALS AND METHODS

### 2.1 Source population

The Clinical Registry Investigating Bardet–Biedl Syndrome (CRIBBS) is an open‐enrolling international disease registry and data repository designed to follow longitudinal health outcomes of individuals with BBS (ClinicalTrials.gov: NCT02329210). As of February 2021, there were 607 individuals from 39 countries enrolled in CRIBBS. Individuals meeting established diagnostic criteria^16^ and/or genetic confirmation of BBS were included in the present study. Prior to inclusion in this Marshfield Clinic Health System Review Board‐approved registry, informed consent was obtained from those enrolled or their legal guardians. A senior research coordinator collected health information during annual health interviews with study participants. Protected health information (PHI) was gathered from medical records obtained in compliance with the Health Insurance Portability and Accountability Act. Available imaging studies, genetic testing, and laboratory studies were obtained from the health care providers of the study participants.

### 2.2 Measures

#### 2.2.1 Kidney failure

KF was defined as irreversible loss of kidney function with an estimated glomerular filtration rate (eGFR) < 15 ml/min/1.73 m^2^ consistent with stage 5 CKD.^28^ In children under 18 years‐of‐age, the Bedside Schwartz equation was used to calculate eGFR.^29^ The eGFR was calculated in adults using the Modification of Diet in Renal Disease equation.^30^ The KF age of onset was preferentially determined based on serum creatinine values, but when not available was determined based on institution of renal replacement therapy.

#### 2.2.2 Genetic variants

The BBS diagnosis was supported by genetic testing in 364 CRIBBS participants. There were 18 different BBS genes represented in the CRIBBS cohort, listed according to descending frequency: *BBS1*, *BBS10*, *BBS2*, *BBS7*, *MKKS*, *BBS9*, *BBS12*, *SDCCAG8*, *BBS4*, *ARL6*, *TTC8*, *BBS5*, *CEP164*, *BBIP1*, *CEP290*, *IFT140*, *IFT172*, *TTC21B*. Heterozygous missense variants of uncertain significance were considered putative variants in subjects meeting BBS clinical diagnostic criteria. Genetic variants were classified as either missense or truncating variants as previously described.^31^ CRIBBS participants with genetic diagnoses were classified by variant type into three groups: two truncating variants, one truncating variant/one missense variant, and two missense variants.^31^ Genes were also classified by the role in primary cilia function played by the proteins they encoded. Proteins were classified as BBSome, chaperonin‐like, or other BBS proteins.

### 2.3 Co‐morbidities and KF

Insulin dependent diabetes mellitus (T1DM) was not present in the KF group. Onset of insulin resistant diabetes mellitus (T2DM) was determined based on participant recall of age at diagnosis and confirmed by medical records when available. Prematurity as a risk for KF was examined. Participants born at 34 weeks of gestation or earlier were compared for KF prevalence in both cohorts. Body mass index and presence of hypertension prior to KF were not collected from CRIBBS participants due to potential recall inaccuracy. CRIBBS data resources do not provide comprehensive information on microalbuminuria and metabolic syndrome preventing analysis of these risk factors.

### 2.4 Statistical analysis

Descriptive summaries were presented to characterize the BBS cohort, with genetic summaries necessarily limited to the sub‐cohort (364/609) who had genetic confirmation. Group comparisons were based on Fisher's exact test for 2 × 2 tables and on Pearson's Chi‐square for larger categorical comparisons. The primary statistical analyses were time‐to‐event analyses of mortality and KF incidence. The time scale in these analyses was patient age, with censoring at age of last interview/contact for those alive, not diagnosed. The Kaplan–Meier^32^ method was used for basic estimates of the cumulative KF event rate, with confidence limits based on the log–log transformation and comparisons of survival based upon the log‐rank test. Proportional hazards regression with diabetes as a time‐varying covariate was used to assess changes in the risk of KF with development of diabetes.^33^

## 3 RESULTS

### 3.1 Prevalence of KF and mortality in BBS

**FIGURE 1:**
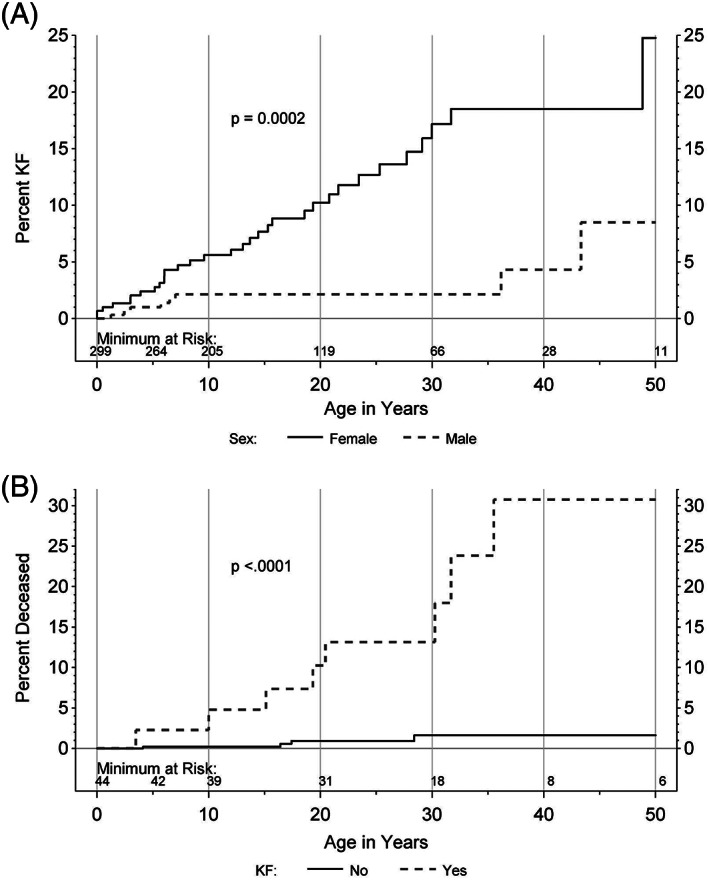
(A) Kidney failure (KF) prevalence by age and sex; (B) All‐cause mortality risk in KF population

Using the largest international registry and data repository for the renal ciliopathy of BBS, we identified KF in 44 of the 607 participants, thus documenting a prevalence of KF in 7.2% of the study group. The age of diagnosis of KF, age of death, sex, and clinical features of BBS are provided in Table 1. The sex distribution in CRIBBS participants is nearly equal (307 males: 300 females); however, we observed a disproportionate prevalence of KF, with females making up 77.3% of the KF group (34/44) compared to 22.7% males (10/44, *p* = 0.0001). Figure 1A provides estimates of the cumulative KF event risk based on age and sex, illustrating the increased risk of KF in females beginning in childhood and persisting into adulthood. Of the CRIBBS participants, 22 are deceased. Kidney failure and death were present in 11 individuals. Additionally, seven infants with severe genitourinary and other anomalies died within the first week of life (data not shown). Figure 1B presents all‐cause mortality risk in those with and without KF.

**TABLE 1:** Primary and Secondary BBS Features of CRIBBS registrants with KF

ID	Sex	ESRD onset (years)	Death (years)	RD	PD	Obesity (BMI)	LD	Hypogonadism in males	GU anomalies	Secondary features
137	M	6	−	+	−	+ (22.15)	+	−		SD^a^, Other eye problems^b^, DD, Polyuria/dipsia, Ataxia, Dental/palate anomalies^c^
168	F	16	−	+	+	+ (20.7)	+	N/A		SD, Brachy/syndactyly, DD, Polyuria/dipsia, Ataxia, Dental/palate anomalies
251	F	28	+ (30)	+	+	+ (36.44)	+	N/A		SD, Other eye problems, Brachy/syndactyly, DD, Polyuria/dipsia, Ataxia
25	F	8	−	+	+	+ (41.8)	+	N/A		SD, Other eye problems, Brachy/syndactyly, DD, Polyuria/dipsia, LVH, Hepatic fibrosis
46	F	8	−	+	+	+ (41.8)	+	N/A		SD, Brachy/syndactyly, DD, Ataxia, Dental/palate anomalies
62	F	2	+ (10)	+	−	+ (not available)	+	N/A		SD, Other eye problems, Brachy/syndactyly, DD, Ataxia
63	F	22	−	+	−	+ (43.3)	−	N/A	Urogenital sinus	Other eye problems, Brachy/syndactyly, Ataxia, Dental/palate anomalies
65	F	2	−	+	+	+ (35.9)	+	N/A		SD, Other eye problems, Ataxia, Mild spasticity, Dental/palate anomalies
77	M	28	−	+	+	+ (30.65)	+	+		SD, Other eye problems, Brachy/syndactyly, DD, Polyuria/dipsia, Ataxia, Dental/palate anomalies
87	F	22	−	+	+	+ (45.87)	+	N/A	Urogenital sinus	Other eye problems, Brachy/syndactyly, T2DM, Dental/palate anomalies
121	F	13	−	+	+	+ (60.73)	+	N/A		Brachy/syndactyly, DD, Polyuria/dipsia, Ataxia, Dental/palate anomalies
257	M	65	+ (70)	+	+	+ (41.5)	+	+		Other eye problems, Polyuria/dipsia, Ataxia, T2DM
361	F	49	−	+	−	+ (38.57)	+	N/A		Other eye problems, DD, Ataxia, T2DM, Dental/palate anomalies
531	M	0	−	+	−	+ (30.04)	+	+		SD, Other eye problems, Brachy/syndactyly, DD, Ataxia, Dental/palate anomalies, hearing loss
534	F	21	−	+	−	+ (55.39)	+	N/A		SD, Brachy/syndactyly, DD, Polyuria/dipsia, Ataxia, Dental/palate anomalies
118	F	29	−	+	−	+ (45.24)	+	N/A		Brachy/syndactyly
119	F	25	−	+	+	+ (46.4)	+	N/A		Brachy/syndactyly, Polyuria/dipsia
64	M	54	+ (51)	+	+	+ (28.9)	+	−		Other eye problems, Brachy/syndactyly, Polyuria/dipsia, Ataxia, T2DM, Dental/palate anomalies, CHD, Cardiac transplant
397	F	55	−	+	+	+ (43.42)	+	N/A		Other eye problems, Brachy/syndactyly, Ataxia, T2DM, Dental/palate anomalies, Hepatic fibrosis
756	M	47	−	+	+	+ (29.7)	+	+		SD, Other eye problems, Brachy/syndactyly, DD, Ataxia, Dental/palate anomalies, LVH, CHD, Cardiac transplant
826	F	28	−	+	+	+ (40.39)	+	N/A		SD, Brachy/syndactyly, DD, Dental/palate anomalies, LVH
360	F	2	−	+	+	‐ (20.74)	−	N/A		Other eye problems, Brachy/syndactyly
621	F	8	−	+	−	+ (23.04)	+	N/A		SD, Brachy/syndactyly, DD, Polyuria/dipsia, Ataxia, Dental/palate anomalies
639	F	0	−	−	+	+ (27.54)	+	N/A	Vesicoureteral reflux	SD, Other eye problems, Developmental delay, LVH
237	F	19	−	+	+	+ (34.72)	+	N/A		SD, Other eye problems, Brachy/syndactyly, DD, Ataxia, Dental/palate anomalies
701	F	18	−	+	+	+ (30.49)	+	N/A	Urogenital sinus	SD, Other eye problems, Brachy/syndactyly, Dental/palate anomalies
171	F	10	−	+	−	+ (44.06)	+	N/A		SD, Other eye problems, Brachy/syndactyly, Developmental delay, Mild spasticity
172	F	4	+ (19)	+	+	+ (39.32)	+	N/A		SD, Other eye problems, Brachy/syndactyly, DD, Ataxia, Dental/palate anomalies
680	F	15	−	+	+	+ (31.93)	+	N/A		SD, Other eye problems, DD, Ataxia, Dental/palate anomalies
430	F	21	−	+	−	+ (31.64)	+	N/A		SD, Other eye problems, Brachy/syndactyly, DD, Ataxia, Dental/palate anomalies
182	M	6	−	+	−	+ (37.4)	+	+		SD, DD, Polyuria/dipsia, Ataxia
183	M	2	+ (3.5)	Unknown^a^	−	+ (not available)	+	−	Hypospadias	SD, DD, Ataxia, infancy onset of respiratory disease
591	F	6	−	+	−	+ (29.3)	+	N/A		SD, Brachy/syndactyly, DD, Ataxia
706	F	12	−	+	−	+ (41.21)	−	N/A		Other eye problems, Ataxia
744	F	6	−	+	−	+ (31.64)	+	N/A		SD, DD
127	F	5	−	+	−	+ (44.13)	−	N/A		Other eye problems, Brachy/syndactyly, ataxia, Hepatic fibrosis, Spasticity, Liver transplant
67	F	0	−	+	+	+ (28.31)	−	N/A	Urogenital sinus	Other eye problems, Brachy/syndactyly, DD, Polyuria/polydipsia, Dental/palate anomalies,
112	F	13	−	+	+	+ (49.53)	+	N/A	Urogenital sinus	SD, Other eye problems, Brachy/syndactyly, DD, Ataxia, Dental/palate anomalies
165	F	3	+ (15)	+	+	+ (32.9)	+	N/A		SD, DD
239	F	6	−	+	+	+ (47.3)	+	N/A		Brachy/syndactyly, DD, Polyuria/polydipsia, Ataxia
297	F	30	+ (36)	+	−	+ (32.72)	+	N/A		SD, Brachy/syndactyly, DD, Ataxia
395	F	57	−	+	+	+ (41.02)	−	N/A		T2DM
403	M	3	−	+	−	+ (23.8)	+	+		SD, Other eye problems, Brachy/syndactyly, DD, Ataxia, Dental/palate anomalies
412	M	6	+ (20)	+	+	+ (46.8)	+	+	Hypospadias	DD, dental anomalies

*Note*: Clinical Signs: +, present; −, absent.

Abbreviations: CHD, congenital heart disease; DD, developmental delay; ESKD, end stage kidney disease; LD, learning disability; LVH, left ventricular hypertrophy; PD, polydactyly; RD, retinal dystrophy; SD, speech disorder/delay; T2DM, diabetes mellitus type 2.

^a^ Not evaluated for retinal degeneration prior to death at 3 years of age.

^b^ Includes strabismus, cataracts, and/or astigmatism.

^c^ Includes dental crowding, hypodontia, small roots, and/or high arched palate.

### 3.2 Gene variants and KF

**FIGURE 2:**
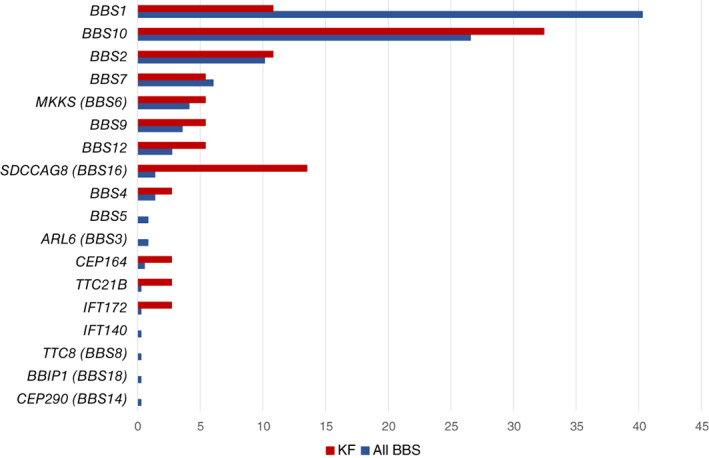
Gene frequency in kidney failure (KF) cohort compared with all CRIBBS participants [Colour figure can be viewed at wileyonlinelibrary.com]

We report 37 individuals with KF who were homozygous or compound heterozygous carriers of 40 different putative disease‐causing variants. (Table 2) These variants were in 12 different BBS genes—*BBS1*, *BBS2*, *BBS4*, *BBS6*, *BBS7*, *BBS9*, *BBS10*, *BBS12*, *SDCCAG8*, *CEP164*, *IFT172*, *and TTC21B*—in individuals with KF, including 10 novel variants (Table 2). Genetic testing was unavailable in seven individuals. Whole exome sequencing did not identify disease‐causing variants in Case 403. There were 18 different BBS genes identified in the CRIBBS cohort. The percentage of individuals with homozygous or compound heterozygous variants in each gene compared to genetically confirmed KF subjects is shown in Figure 2.

**TABLE 2:** Genetic characterization of CRIBBS registrants with kidney failure

Case #	Family #	Ancestry	Gene	DNA variation	Predicted effect	Classification*	Reference
137	1	European	*BBS1*	c.1169 T > G	p.Met390Arg	Pathogenic	[34]
				c.1285C > T	p.Arg429*	Pathogenic	[35]
168	2	European	*BBS1*	c.1169 T > G	p.Met390Arg	Pathogenic	[34]
				c.1169 T > G	p.Met390Arg	Pathogenic	
239	3	Iranian	*BBS1*	c.1432del	p.Leu478Cysfs*101	Pathogenic	Novel^a^
				c.1432del	p.Leu478Cysfs*101	Pathogenic	
251	4	European, Native American	*BBS1*	c.1169 T > G	p.Met390Arg	Pathogenic	[34]
				c.1169 T > G	p.Met390Arg	Pathogenic	
25	5	Mexican, European	*BBS10*	c.271dup	p.Cys91Leufs*5	Pathogenic	[36]
				c.271dup	p.Cys91Leufs*5	Pathogenic	
46	6	European	*BBS10*	c.271dup	p.Cys91Leufs*5	Pathogenic	[36]
				c.271dup	p.Cys91Leufs*5	Pathogenic	
62	7	European, Native American	*BBS10*	c.271dup	p.Cys91Leufs*5	Pathogenic	[36]
				c.271dup	p.Cys91Leufs*5	Pathogenic	
63	7	European, Native American	*BBS10*	c.271dup	p.Cys91Leufs*5	Pathogenic	[36]
				c.271dup	p.Cys91Leufs*5	Pathogenic	
65	8	European	*BBS10*	c.310_311del	p.Glu104Lysfs*7	Pathogenic	[20]
				c.271dup	p.Cys91Leufs*5	Pathogenic	[36]
77	9	European	*BBS10*	c.1677C > A	p.Tyr559*	Pathogenic	[20]
				c.271dup	p.Cys91Leufs*5	Pathogenic	[36]
87	10	European	*BBS10*	c.909_912del	pSer303Argfs*3	Pathogenic	[36]
				c.271dup	p.Cys91Leufs*5	Pathogenic	[36]
121	11	European	*BBS10*	c.590A > G	p.Tyr197Cys	Pathogenic	[36]
				c.271dup	p.Cys91Leufs*5	Pathogenic	[36]
257	12	European	*BBS10*	c.145C > T	p.Arg49Trp	Pathogenic	[36]
				c.1804G > A	p.Val602Leu	Pathogenic	Novel^a^
361	13	European	*BBS10*	c.271dup	p.Cys91Leufs*5	Pathogenic	[36]
				c.271dup	p.Cys91Leufs*5	Pathogenic	
531	14	Unknown	*BBS10*	c.271dup	p.Cys91Leufs*5	Pathogenic	[36]
				c.271dup	p.Cys91Leufs*5	Pathogenic	
534	15	European	*BBS10*	c.1184dup	His395Glnfs*14	Pathogenic	[37]
				c.2119_2120del	p.Val707*	Pathogenic	[36]
118	16	Pakistani, Indian	*BBS12*	c.1438del	p.Asp480Metfs*3	Pathogenic	[20]
				c.1438del	p.Asp480Metfs*3	Pathogenic	
119	16	Pakistani, Indian	*BBS12*	c.1438del	p.Asp480Metfs*3	Pathogenic	[20]
				c.1438del	p.Asp480Metfs*3	Pathogenic	
64	17	European	*BBS2*	c.1895G > C	p.Arg632Pro	Pathogenic	[38]
				c.311A > C	p.Asp104Ala	Pathogenic	[38]
397	18	European	*BBS2*	c.823C > T	p.Arg275*	Pathogenic	[38]
				c.823C > T	p.Arg275*	Pathogenic	
756	19	European	*BBS2*	c.613‐1G > C		Pathogenic	[39]
				c.535C > T	p.Leu179Phe	VUS	Novel^a^
826	20	European	*BBS2*	c.565C > T	p.Arg189*	Pathogenic	[40]
				c.653G > A	p.Gly218Asp	VUS	Novel^a^
360	21	European	*BBS4*	c.220 + 1G > C		Pathogenic	[34]
				c.513 T > A	p.Tyr171*	Pathogenic	[41]
621	22	European	*MKKS* (*BBS6*)	c.110A > G	p.Tyr37Cys	Pathogenic	[42]
				c.592_593del	p.Lys198Glufs*23	Pathogenic	[43]^d^
639	23	European, Native American	*MKKS* (*BBS6*)	c.121G > C	p.Gly41Arg	VUS	[20]
				Exon 4–5 deletion		Pathogenic	Novel^a^
237	24	Laotian	*BBS7*	c.389_390del	p.Asn130Thrfs*4	Pathogenic	[44, 45]
				c.389_390del	p.Asn130Thrfs*4	Pathogenic	
701	25	Laotian	*BBS7*	c.389_390del	p.Asn130Thrfs*4	Pathogenic	[44, 45]
				c.389_390del	p.Asn130Thrfs*4	Pathogenic	
171	26	European, Armenian	*BBS9*	c.1877_1880del	p.Lys626Argfs*22	Pathogenic	[46]
				c.2007_2008dup	p.Ala670Glufs*13	Pathogenic	[47]
172	26	European, Armenian	*BBS9*	c.1877_1880del	p.Lys626Argfs*22	Pathogenic	[46]
				c.2007_2008dup	p.Ala670Glufs*13	Pathogenic	[47]
680	27	Mexican	*CEP164*	c.4001G > A	p.Trp1334*	Pathogenic	Novel^a^
				Exon 29–30 deletion		Pathogenic	Novel^a^
430	28	European	*IFT172*	c.5068G > C	p.Gly1690Arg	VUS	[48]^e^
				c.5179 T > C	p.Cys1727Arg	Pathogenic	[49, 50]^b^
182	29	European	*SDCCAG8* (*BBS16*)	c.696 T > G	p.Tyr232*	Pathogenic	[51]
				Exon 6–8 deletion		Pathogenic	Novel^a^
183	29	European	*SDCCAG8* (*BBS16*)	c.696 T > G	p.Tyr232*	Pathogenic	[52]
				Exon 6–8 deletion		Pathogenic	Novel^a^
591	30	Pakistani	*SDCCAG8* (*BBS16*)	c.1221 + 2 T > A		Pathogenic	Novel^a^
				c.1221 + 2 T > A		Pathogenic	
706	31	European	*SDCCAG8* (*BBS16*)	c.696 T > G	p.Tyr232*	Pathogenic	[51]
				c.740 + 356C > T		Likely Pathogenic	[51]
744	32	European	*SDCCAG8* (*BBS16*)	c.1120C > T	p.Arg374*	Pathogenic	[51]
				c.1120C > T	p.Arg374*	Pathogenic	
127	33	European	*TTC21B*	c.626C > T	p.Pro209Leu	Pathogenic	[25]
				c.1320del	p.Phe440Leufs*4	Likely Pathogenic	[52]^c^
67	34	European	Unknown				
38	35	European	Unknown				
112							
165	36	European	Unknown				
239	37	Iranian	Unknown				
297	38	European	Unknown				
395	39	European	Unknown				
03	40	European	Unknown^f^				
412	41	European	Unknown				

*Note*: * Indicates classification according to ACMG guidelines.

Abbreviations: CRIBBS, Clinical Registry Investigating Bardet–Biedl Syndrome; KF, kidney failure; VUS, variant of uncertain significance.

^a^ Not previously reported in literature or ClinVar, to our knowledge.

^b^ Reported in multiple patients with asphyxiating thoracic dystrophy and Mainzer‐Saldino syndrome.

^c^ Reported in short rib‐polydactyly syndrome, type 4.

^d^ Identified in patient with nephronophthisis.

^e^ Identified in patient with retinitis pigmentosa and short‐rib thoracic dysplasia syndrome.

^f^ Non‐diagnostic WES.

*BBS1* is the most common BBS gene in North American and European populations, accounting for 40.3% of CRIBBS participants with genetic confirmation, but it was identified in only 10.8% of the genetically confirmed KF cohort. *BBS10*, the second most common identified gene in the CRIBBS cohort (26.6%), was present in 32.4% of the KF population with genetic confirmation. Notably, there were five CRIBBS registrants with *SDCCAG8* variants, and all five of these individuals developed KF before 13 years‐of‐age. Polydactyly, a primary feature of BBS, was absent in all *SDCCAG8* cases (Table 1).

**FIGURE 3:**
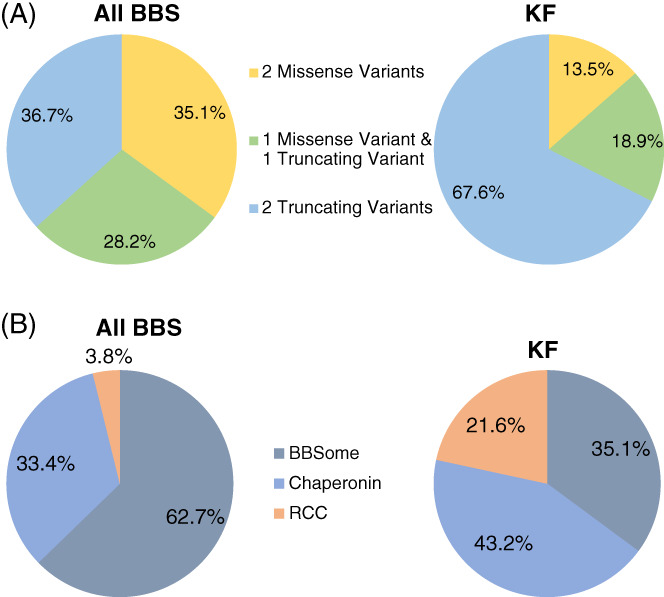
Distribution of CRIBBS participants with molecular diagnosis by (A) gene variant and (B) gene group [Colour figure can be viewed at wileyonlinelibrary.com]

Comparisons of variant type prevalence in the KF cohort and total CRIBBS population are shown in Figure 3A. Only individuals with two putative variants in one BBS gene were included in this analysis. In the CRIBBS cohort, individuals with two truncating variants make up the largest group (36.7%), followed by individuals with two missense variants (35.1%), and then individuals with one truncating and one missense variant (28.2%). In the KF group, individuals with two truncating variants made up an even larger portion (67.6%), followed by individuals with one truncating and one missense variant (18.9%), and then individuals with two missense variants (13.5.%). This variant type distribution for individuals with KF was significantly different from for individuals without KF (*p* = 0.0002).

### 3.3 KF in BBSome versus chaperonin‐like versus other BBS genes

**FIGURE 4:**
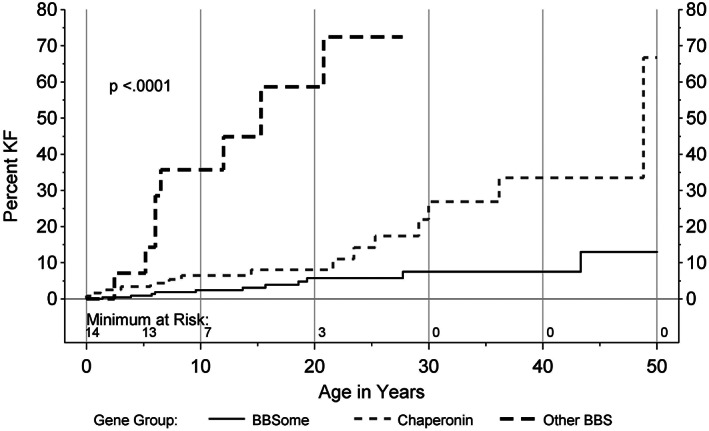
Kidney failure (KF) incidence by age and protein group

There were 229 CRIBBS participants with variants in *BBS1*, *BBS2*, *BBS4*, *BBS5*, *BBS7*, *TTC8*, *BBS9*, and *BBIP1* genes, which are known to assemble into the BBSome.^18^ There were 121 participants with variants in *MKKS*, *BBS10*, and *BBS12* genes, which make up the chaperonin‐like complex.^19^, ^20^ There were 14 participants with pathogenic variants in *ARL6*, *CEP164*, *CEP290*, *SDCCAG8*, *IFT140*, *IFT172*, *and TTC21B*, collectively designated as other BBS genes. KF incidence across protein groups is shown in Figure 3B. The incidence of KF is increased in the other BBS genes (*p* < 0.001). By age 20, 58.7% (95% confidence limits 31.5%–87.3%) of individuals with variants in other BBS genes developed KF, as compared to 5.8% (3.0%–11.2%) in BBSome genes, and 8.1% (4.0%–15.8%) in chaperonin genes (Figure 4).

### 3.4 Structural uropathies

A summary of uropathies present in the CRIBBS participants is shown in Table 3. Diverse lower urinary tract anomalies were present in the CRIBBS cohort. One or more uropathies was identified in 98 (16.1%) of participants. The prevalence of uropathies was similar in both males (45.9%) and females (54.1%).

**TABLE 3:** Structural uropathies in ESKD and non‐ESKD participants

All				Females				Males			
	KF	Non‐KF	*p* value		KF	Non‐KF	*p* value		KF	Non‐KF	*p* value
VUR	2.3% (1/44)	6.2% (35/563)	0.504	Urogenital Sinus	14.7% (5/34)	6.0% (16/266)	0.074	PUV	0.0% (0/10)	0.3% (1/297)	1.0
EBS	0.0% (0/44)	0.7% (4/563)	1.0	Vaginal Atresia	0.0% (0/34)	3.8% (10/266)	0.610	Chordee	0.0% (0/10)	5.1% (15/297)	1.0
								Hypo‐ or epispadius	20.0% (2/10)	6.1% (18/297)	0.133

*Note*: *p* values are based on Fisher's exact test.

Abbreviations: EBS, Eagle‐Barrett syndrome; KF, kidney failure; PUV, posterior urethral valves; VUR, vesicoureteral reflux.

### 3.5 Comorbid conditions present prior to KF

Insulin dependent diabetes mellitus (T1DM) was not present in the KF cohort and was reported in four individuals (0.7%) in the non‐KF cohort. Insulin resistant diabetes mellitus (T2DM) was present prior to KF in 6 KF subjects (13.6%) and in 49 non‐KF subjects (8.7%). T2DM did not show significance in a proportional hazards model for time to KF with T2DM as a time‐varying covariate (*p* = 0.35. hazard ratio 1.63), although statistical power was limited by small numbers. Two individuals were born before 35 weeks gestation in the KF cohort and 30 non‐KF individuals were born before 35 weeks gestation (*p* = 0.49). The prevalence, duration, and severity of microalbuminuria, hypertension, obesity or metabolic syndrome, prior to KF potentially contributing to kidney failure were not available in the CRIBBS database.

## 4 DISCUSSION

Chronic kidney disease is a dominant disease in BBS resulting in increased morbidity and premature death. Premature death is uniquely increased in individuals with KF regardless of etiology.^53^, ^54^ The present study systematically examines genetics of the syndrome, providing insight into genotype and phenotype correlations with KF. At least 26 different genes have been identified as causative genes in BBS, with many being private familial pathogenic and likely pathogenic variants in the identified genes.^2^, ^3^, ^4^ The implications of the genetic variants and attendant risk of KF is largely unexplored. Two previous studies identified more severe kidney disease associated with *BBS2*, *BBS10*, and *BBS12* in a United Kingdom cohort of children and adults and *BBS6*, *BBS10*, and *BBS12* in a French cohort of adults^11^, ^13^ Utilizing the largest international natural history registry, our findings provide critical insight and advance opportunities to explore the proteomics and metabolomics in a model renal ciliopathy.^6^, ^15^ We proffer new insight in the complexity and phenotypic diversity of ciliopathies reporting biallelic variants in *TTC21B* in an individual meeting diagnostic criteria for BBS and experiencing both kidney and liver failure requiring combined organ transplantation. Biallelic variants in *TTC21B* have not be previously identified in BBS but are causative for both nephronophthisis (NPHP) and Juene Asphyxiating Thoracic Dystrophy.^25^ *TTC21B* in *trans* with other ciliopathy genes is postulated to contribute to the severity/penetrance of the primary ciliopathy.^25^ Our findings identify gene variants previously unreported or rarely reported in BBS. Structural anomalies of the lower urinary tract that may contribute to the risk of KF have been investigated. Interestingly, the diversity of uropathies in individuals with BBS is expanded in this report, providing new awareness the pleiotropic features of BBS. Notably, the study provides insight into the value of registries in advancing research in rare diseases.

The present study confirms that severe kidney disease is more commonly identified in patients with pathogenic variants in *BBS10* compared to *BBS1*. Pathogenic variants in *BBS10* are predominantly truncating variants, while missense variants are most common in *BBS1*. Increased risk of cardiovascular disease and childhood obesity have previously been associated with truncating variants in BBS.^13^, ^31^ We have documented in this report that KF is an increased risk in individuals with truncating variants in multiple genes, including but not limited to *BBS10*. The age of KF identification was earlier in life in individuals with truncating variants. The reduced severity of kidney disease in individuals with missense variants could be explained by some level of residual protein function. Interestingly, one individual with two missense variants in *BBS10* reached KF in the seventh decade of life, significantly later than other individuals with truncating variants in *BBS10*. Additionally, one individual with a homozygous truncating variant in *BBS1* and another individual that was a compound heterozygote with one truncating variant and one missense variant in *BBS1* developed KF at 6 years‐of‐age, while those with missense variants in *BBS1* rarely developed KF—only two out of 107 individuals with biallelic missense variants in *BBS1*. Similar to reports in the most common renal ciliopathy of autosomal dominant polycystic kidney disease, we identified individuals with lower risk BBS genotypes with early onset KF, suggesting atypical early onset KF must be considered in individuals with missense variants. The early onset of KF in individuals with low‐risk BBS variants suggests additional genetic modifiers may have influence in these cases. Discordant disease severity is recognized in renal diseases including other renal ciliopathies without identified mechanisms.^55^, ^56^ We confirm and advance the finding that truncating variants in *SDCCAG8*, a gene represented in <1.4% of the CRIBBS population, has a highly predictive risk of early onset KF.^51^, ^57^ Similar to previous reports, polydactyly was not present in individuals with *SDCCAG8*, suggesting increased awareness of the importance of genetic testing for BBS in children with severe CKD without the primary feature of polydactyly.^51^, ^57^

The BBS genes and their association with KF have been examined in this report using three distinct categories, namely the BBSome, the chaperonin‐like genes, and other BBS genes. Pathogenic variants in the chaperonin proteins are postulated to have disproportionate negative impact on BBS phenotype.^19^, ^20^ Our report does not confirm that chaperonin‐like proteins collectively pose increased risk of KF. This is consistent with our previous finding that there were no differences in body mass index *z*‐score for any age group between chaperonin‐like and BBSome pathogenic variants.^31^ The *ARL6* protein recruits the BBSome to the ciliary membrane and is not associated with KF in this report or in other studies.^11^, ^13^, ^26^ The other BBS genes group has varied roles, including regulation of BBSome‐centriole docking, formation of a transition zone at the plasma membrane, and primary cilia intraflagellar vesicle transport.^2^, ^58^ The genes are identified in overlapping syndromes including NPHP, JATD, Senior‐Løken syndrome, Joubert syndrome, short rib‐polydactyly syndrome, and Mainzer‐Saldino syndrome.^2^, ^3^, ^5^, ^30^, ^59^ We have identified significant risk for early onset KF associated with the other BBS genes. This report gives additional insight on the previously reported BBS genes of *CEP164*, *IFT172*, *SDCCAG8*,^4^, ^48^, ^49^, ^50^, ^51^ and *TTC21B*, a new BBS candidate gene. The ongoing discovery of causative genes for BBS strongly supports the use of comprehensive BBS sequencing panels, including consideration of exome and/or genome sequencing in individuals with unexplained CKD or uropathies.

Anatomic abnormalities are common in BBS including cardiovascular, gastrointestinal, craniofacial, orthopedic, and respiratory tract anomalies.^16^, ^26^, ^59^, ^60^ Situs inversus totalis and heterotaxy are at least 170‐fold more common compared to the general population.^60^ In the present study, we documented a wide variety of genitourinary (GU) anomalies. Some of the GU findings have been reported in case reports, case series, and disease surveys.^16^, ^61^, ^62^ A recent meta‐analysis study used previously published reports to characterize the prevalence of GU findings.^26^ The present report employs CRIBBS to examine the diversity of GU anomalies and reports both the incidence of the findings as well as previously unreported findings. We identified four children with Eagle Barrett syndrome, a condition previously unreported in BBS.^61^, ^62^, ^63^, ^64^ The role of GU anomalies in KF is particularly important. In the general pediatric population KF is disproportionately represented in males during childhood and primarily associated with bladder outlet obstruction.^63^, ^64^ The present study identifies urogenital sinus and male urethral anomalies in some KF patients, but structural anomalies of the lower urinary tract are not increased compared to the general BBS population. This suggests KF was primarily due to intrinsic kidney disease rather than obstructive uropathy or vesicoureteral reflux. Although urogenital sinus is only identified in females with *BBS2*, *BBS7*, and *BBS10*, there is insufficient evidence that the complex GU anomaly is uniquely associated with specific genetic variants.

Rare disease registries provide unique opportunities to evaluate genotype and phenotype correlations in rare diseases associated with KF.^65^, ^66^ The findings in this report likely have validity for genetic counseling regardless of patient ancestry. We report female sex as a risk factor for KF in BBS. Forsythe et al. did not observe a similar finding (personal communications). Interestingly, similar sex differences were not observed in previous CRIBBS‐based studies examining childhood obesity^31^ and sleep and physical activity patterns.^67^ Our study has some limitations. Potential risk factors for CKD progression including untreated or suboptimal treatment of hypertension, albuminuria, and metabolic syndrome cannot be comprehensively evaluated based on registry data. Examination of obesity as an independent risk for KF in BBS will require future investigation. We have previously reported that obesity was not disproportionately severe in renal transplant recipients with BBS compared to the CRIBBS cohort.^17^ Unfortunately, genetic testing is not available in all CRIBBS participants, including seven individuals with KF. One individual did have whole exome sequencing without identified pathogenic variants despite meeting diagnostic criteria for BBS. This is not uncommon in individuals with BBS. Confirmed biallelic pathogenic variants are not identified in 20% of individuals meeting diagnostic criteria.^27^ It would also be ideal to examine the role of additional ciliopathy genes—mutational burden—contributing to the BBS KF phenotype. Targeted testing or limited gene panels are commonly utilized in individuals with suspected BBS, and exome sequencing or larger ciliopathy panels are not available in all CRIBBS participants. Furthermore, we have not examined other genetic modifiers, protein–protein interactions, or environmental factors that may contribute to disease variability.

We conclude that KF poses a significant risk factor for premature morbidity in individuals with BBS. Furthermore, biallelic truncating variants are predominantly associated with KF, although KF can be observed in individuals with missense variants, including individuals with *BBS1*, suggesting the importance of vigilant monitoring of kidney function in all individuals with BBS. We have identified high‐risk individuals meeting diagnostic criteria for BBS and possessing gene variants associated with other renal ciliopathies, documenting the importance of comprehensive genetic evaluation including consideration of exome or genome sequencing in individuals with atypical features of CKD.

## CONFLICT OF INTEREST

RH is a consultant for Rhythm Pharmaceuticals and Axovia Therapeutics, LLC and principal investigator for the Setmelanotide Phase 2 Treatment Trial in Patients with Rare Genetic Obesity (ClinicalTrials.gov NCT03013543) and Long Term Extension Trial of Setmelanotide (Clinical Trials.gov NCT03651765) sponsored by Rhythm Pharmaceuticals. JP receives research funding from Rhythm Pharmaceuticals and Axovia Therapeutics. SH received research funding from Rhythm Pharmaceuticals. All other authors report no conflict of interest.

## AUTHOR CONTRIBUTIONS

Jennifer R. Meyer, Jesse G. Richardson, Richard L. Berg, and Robert M. Haws designed the work and wrote the manuscript. Richard L. Berg conducted statistical analysis. Jesse G. Richardson, Anthony D. Krentz, Scott J. Hebbring, and Jennifer R. Meyer conducted clinical and genetic data analysis. Anthony D. Krentz, Scott J. Hebbring, and JP contributed to the interpretation of the results and writing of the manuscript. All authors critically reviewed the manuscript and approved the final version for submission to *Clinical Genetics*.

### PEER REVIEW

The peer review history for this article is available at https://publons.com/publon/10.1111/cge.14119.

## Data Availability

All source data presented in this manuscript are available in the CRIBBS database. Data access and data sharing from CRIBBS are available with Marshfield Clinic Health System‐approved data transfer agreement.
